# Mass Spectrometry-Based Approaches to Understand the Molecular Basis of Memory

**DOI:** 10.3389/fchem.2016.00040

**Published:** 2016-10-14

**Authors:** Arthur H. Pontes, Marcelo V. de Sousa

**Affiliations:** Laboratory of Protein Chemistry and Biochemistry, Department of Cell Biology, University of BrasiliaBrasilia, Brazil

**Keywords:** memory, hippocampus, synapse, long-term potentiation (LTP), mass spectrometry, selected reaction monitoring (SRM), quantitative proteomics

## Abstract

The central nervous system is responsible for an array of cognitive functions such as memory, learning, language, and attention. These processes tend to take place in distinct brain regions; yet, they need to be integrated to give rise to adaptive or meaningful behavior. Since cognitive processes result from underlying cellular and molecular changes, genomics and transcriptomics assays have been applied to human and animal models to understand such events. Nevertheless, genes and RNAs are not the end products of most biological functions. In order to gain further insights toward the understanding of brain processes, the field of proteomics has been of increasing importance in the past years. Advancements in liquid chromatography-tandem mass spectrometry (LC-MS/MS) have enabled the identification and quantification of thousands of proteins with high accuracy and sensitivity, fostering a revolution in the neurosciences. Herein, we review the molecular bases of explicit memory in the hippocampus. We outline the principles of mass spectrometry (MS)-based proteomics, highlighting the use of this analytical tool to study memory formation. In addition, we discuss MS-based targeted approaches as the future of protein analysis.

## Introduction

Memory is one of the most important components of cognition and it refers to the capacity to store and retrieve new information about the physical world (Kandel et al., 2014). Memory emerged very early in the evolutionary history of the nervous system, and has played a central role in helping organisms to adapt and survive the challenges faced in their environments (Emes et al., 2008; Ryan and Grant, 2009). This cognitive process has reached one of its most complex forms in humans, where deficits can have devastating consequences for the individual. In Alzheimer disease, for example, the build-up of misfolded proteins in the brain disrupts the connectivity between nerve cells, leading to memory impairments and dementia (Ross and Poirier, 2004; Musunuri et al., 2014). In Huntington's disease, the expansion of a CAG triplet in the huntingtin gene leads to motor abnormalities and cognitive decline such as memory loss (Paulsen, 2011). As a result, memory has been the focus of intensive investigation in different research fields such as neurobiology, psychology, and anthropology (Zola-Morgan et al., 1986; Rempel-Clower et al., 1996; Tronson and Taylor, 2007).

The first scientific studies attempting to understand the cellular and molecular basis of memory formation date back to the earliest neuroscientists like Santiago Ramón y Cajal, who formulated the idea that information storage occurs by the physical rearrangement in the connections between nerve cells (Mayford et al., 2012; Bailey et al., 2015). To this day, a plethora of studies in organisms ranging from the nematode *Caenorhabditis elegans* to humans have established that this postulation is correct; hence, Ramón y Cajal's hypothesis has been used as a major conceptual framework for the understanding of memory formation (Rongo and Kaplan, 1999; Tsai et al., 2005; Cooke and Bliss, 2006; Groszer et al., 2008). However, a complete comprehension of the mechanisms underlying information storage is still lacking, since (1) we do not know all the molecular processes that happen at the synapses and (2) how they are related to one another.

Several techniques have been employed to address such questions (Tsien et al., 1996b; Wang et al., 2003; Boyden et al., 2005; Whitlock et al., 2006; Grover et al., 2009; Zheng et al., 2015). For instance, restricted gene knockout, which consists in the deletion of a gene by homologous recombination in a specific region or tissue, has been extensively used in the past few years (Gu et al., 1994; Li et al., 1994; Tsien, 2016). One common type of restricted gene knockout exploits the *Cre/loxP* system, where the bacteriophage P1 enzyme *Cre* recombinase catalyzes homologous recombination between the *loxP* recognition sequences (Tsien et al., 1996a). In a typical experiment, a line of mice having a gene of interest flanked by the *loxP* sequences is generated. Next, a second line of transgenic mice is created, where the *Cre* recombinase is expressed under the control of a neural promoter sequence that is activated only in specific brain regions. By crossing the two lines of animals, the targeted gene will be deleted in those cells that express the *Cre* recombinase (McHugh et al., 1996; Gong et al., 2007; Taniguchi et al., 2011). Because this system is restricted to a particular tissue and region, it became a powerful tool to investigate the underlying mechanisms of memory formation. Nevertheless, this assay tends to be extremely laborious, expensive and confined to a specific sequence of interest.

Recently, high-resolution mass spectrometry (MS) has emerged as an alternative to classical assays such as restricted gene knockout. MS has a high-throughput capability and can identify thousands of proteins and their post-translational modifications (PTMs) in a single experiment (Low et al., 2013; Lin et al., 2015). Notably, the power of this analytical tool has been used to identify novel proteins that participate in important cellular activities and disease states (Old et al., 2005; de Godoy et al., 2008; Azimifar et al., 2014; Tyanova et al., 2016). In this review, we present an overview of the cellular processes responsible for memory formation in the hippocampus. In addition, we highlight how MS-based approaches can be used to study the molecular processes encompassing memory formation.

## Molecular basis of explicit memory

Memory has always been of great interest to the scientific community. Early studies on animal models and patients with lesions on specific brain regions revealed that this cognitive function is not an unitary cerebral faculty, but can be organized into at least two general forms: implicit and explicit memory (Barco et al., 2006; Kandel, 2012). Implicit or non-declarative memory stores information that does not rely on conscious effort, like habits and motor skills (Lynch, 2004). This type of memory has been widely studied on the marine mollusk *Aplysia califorica*, and, in mammals, many neural structures have been associated with it, such as the amygdala, the cerebellum, and reflex pathways (Barzilai et al., 1989; Hawkins et al., 2006; Voss and Paller, 2008). Explicit or declarative memory, on the other hand, deals with the storage and recall of knowledge about facts and events, which depend on conscious awareness. Here, the main neural substrates are the neocortex and the hippocampus (Eichenbaum, 2000; Kandel, 2001).

**Figure 1:**
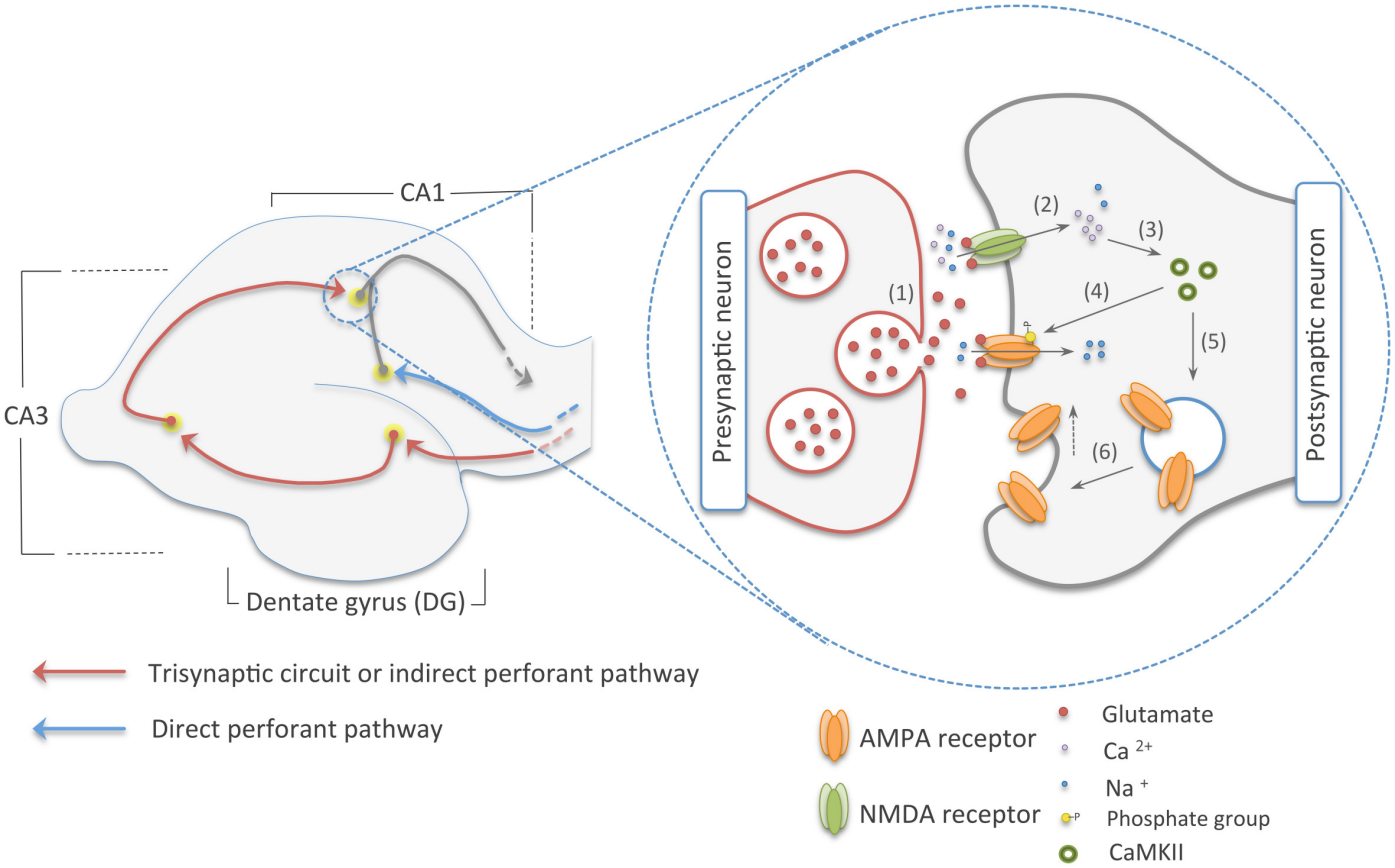
**The two main pathways to the CA1 area of the hippocampus on the left, and early-phase NMDA dependent-LTP on the right**. The red arrows in the picture on the left show the trisynaptic circuit of the hippocampus, where multimodal sensory and spatial information coming from the entorhinal cortex (EC) is relayed to the CA1 area following this route: EC–DG–CA3–CA1. In blue, we illustrated the direct perforant pathway, which directly connects the EC to the CA1 region. On the picture in right, we show an illustration of the early-phase LTP. Here, (1) glutamate from the presynaptic neuron is released into the synaptic cleft. (2) This neurotransmitter reaches ionic channels of the postsynaptic cell causing depolarization of this neuron by the influx on sodium and calcium cations. (3) Calcium, in its turn, activates CaMKII that (4) phosphorylates ionic channels in the PSDs and (5, 6) induces the addition of AMPA receptors to the postsynaptic membrane, increasing synaptic efficiency.

The hippocampus, a seahorse-shaped structure located in the medial temporal lobe of the vertebrate brain, has long been used as a model system to understand the mechanisms of explicit memory formation (Bliss and Collingridge, 1993; Moser et al., 1993; Whitlock et al., 2006). It can be anatomically divided into three distinct regions: the dentate gyrus (DG), the hippocampus proper—composed of the CA1, CA2, and CA3 areas—and the subiculum (Strange et al., 2014). Each of these sections harbors different populations of cells that communicate through two main pathways, namely the direct perforant pathway and the trisynaptic circuit, also known as the indirect perforant pathway (Kandel et al., 2012). The former transmits multimodal sensory and spatial information directly from the entorhinal cortex (EC) to the CA1 area, a major output of the hippocampus. By contrast, in the trisynaptic circuit, information is sent from the EC to the CA1 area following this route: EC–DG–CA3–CA1 (Figure 1) (Neves et al., 2008; Deng et al., 2010; Suh et al., 2011). Experiments carried out in those distinct populations of cells have demonstrated that they work as cellular engrams (i.e., cells that are the loci of information storage) (Ryan et al., 2015; Tonegawa et al., 2015).

The molecular mechanisms underlying memory formation only began to be comprehended after the discovery of long-term potentiation (LTP), which is a form of experience-dependent plasticity and is believed to be the major cellular correlate of learning and memory (Govindarajan et al., 2011; Nabavi et al., 2014). Induction of LTP occurs throughout the hippocampus via distinct signaling cascades, which can differ greatly based on the animal age and region of the hippocampus (Yasuda et al., 2003; Malenka and Bear, 2004). N-methyl-D-aspartate (NMDA)-dependent LTP is the best-characterized type of LTP (Lüscher and Malenka, 2012). This type of LTP occurs at the major excitatory synapses and follows the Hebbian model of neural selectivity, which postulates that synaptic plasticity is dependent on the simultaneous association of pre and postsynaptic neurons (Bi and Poo, 2001; Lu et al., 2001). Furthermore, NMDA-dependent LTP can be divided into two distinct phases: an early phase that facilitates the transmission of action potentials and lasts from minutes to hours, and a late phase, which is dependent on protein synthesis and lasts from hours to a life time (Abel et al., 1997; Govindarajan et al., 2011; Granger et al., 2013).

The early phase of NMDA-dependent LTP, also referred as the induction phase, begins by the release of glutamate from the presynaptic terminal into the synaptic cleft. Glutamate, the main excitatory neurotransmitter in the brain, diffuses through the cleft and reaches the post-synaptic neuron, where it binds to ligand-gated ion channels. The interaction between the neurotransmitter and the ionotropic NMDA receptor results in the influx of Ca^2+^ and Na^+^ into the cell (Schiller et al., 1998; Dingledine et al., 1999; Castillo, 2012). The Na^+^ helps to bring about a depolarization of the postsynaptic neuron that last a few milliseconds, while the Ca^2+^ promotes the activation of protein kinases such as calcium/calmodulin-dependent protein kinase (CaMKII) (Lisman et al., 2012; Lüscher and Malenka, 2012). CaMKII and other kinases prompt the introduction of other ionotropic channels called α-amino-3-hydroxy-5-methyl-4-isoxazolepropionic acid (AMPA) receptors into the postsynaptic density (PSD)—a structure located in the tip of the dendritic spine of postsynaptic cells composed of ionic receptors and a dense network of proteins that regulate synapse strength (Hayashi et al., 2000; Chen et al., 2008; Lu et al., 2010). This traffic of new AMPA receptors to the membrane ultimately leads to an improvement in synaptic communication (Figure 1).

**Figure 2:**
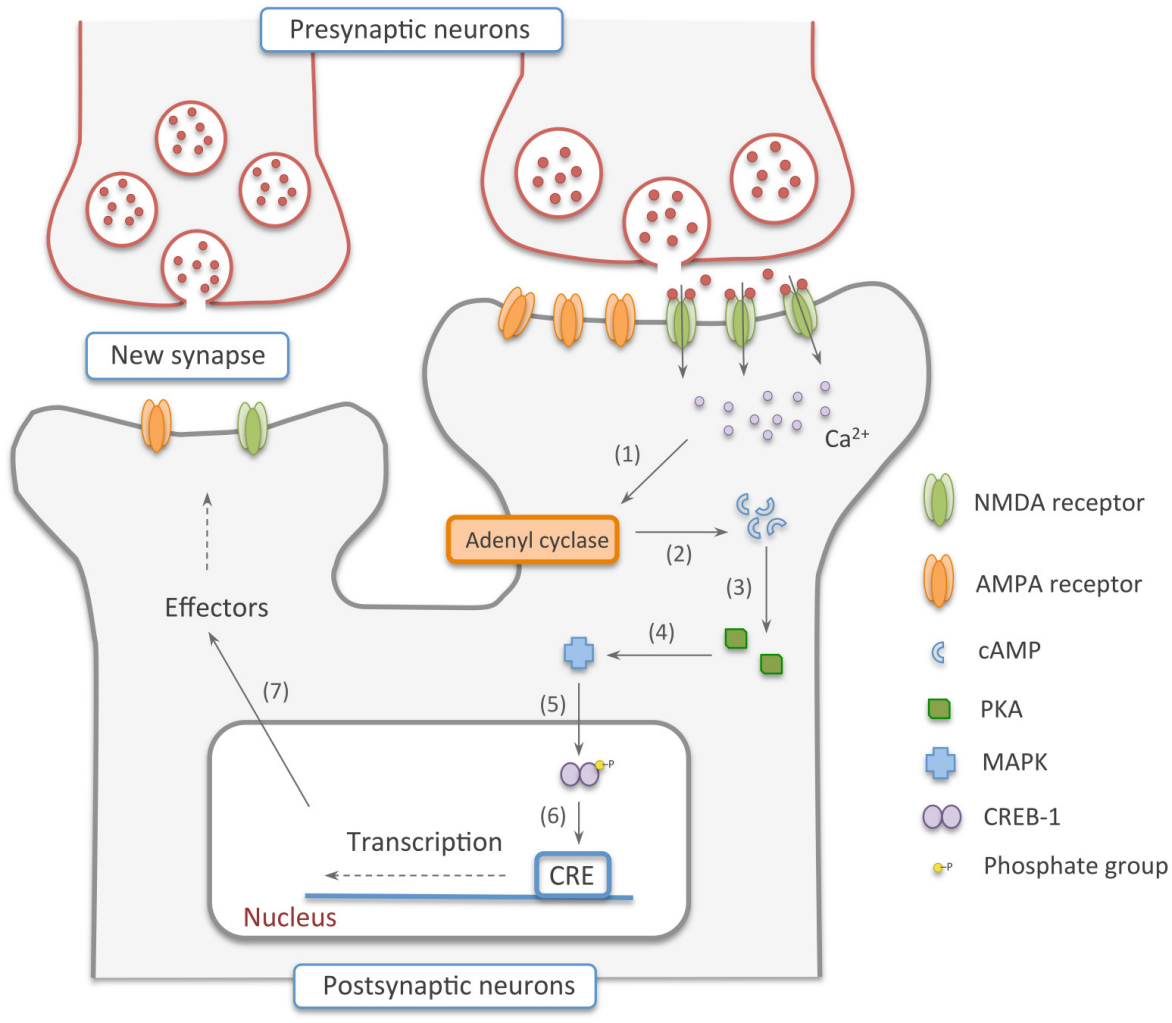
**Late-phase NMDA dependent-LTP**. In this stage, (1, 2) Ca^2+^ ions inside the cell recruit adenyl cyclase to produce cAMP. (3, 4) Cyclic adenosine monophosphate, in turn, activates PKA that switches on MAPK. (5) This kinase is translocated to the nucleus and phosphorylates CREB-1, an important transcription factor. (6, 7) The phosphorylation activates CREB-1, resulting in increased transcription of a number of target genes and their subsequent translation into proteins responsible for the formation of new synaptic connections.

If the activation of the pre and postsynaptic neurons persists for longer periods of time—something that can also be accomplished *in vitro* by repeated stimulation of the cells by high frequency tetanus pulses of 100 Hz, a number of signaling cascades are activated, leading to protein synthesis and synapse rearrangement (Nguyen et al., 1994; Hölscher et al., 1997; Ryan et al., 2015). This is known as the late phase LTP, also called the expression phase. In this stage, the rise in Ca^2+^ ions inside the cell, caused by the constant release of glutamate by the presynaptic cell, induces the increase in the production of cyclic adenosine monophosphate (cAMP) by adenyl cyclase (Wong et al., 1999; Poser and Storm, 2001). cAMP, in turn, activates protein kinase A (PKA) that switches on mitogen activated protein kinase (MAPK) (Abel et al., 1997; Roberson et al., 1999). This kinase is translocated to the nucleus and phosphorylates cAMP response element binding protein 1 (CREB-1), an important transcription factor (Viola et al., 2000; Patterson et al., 2001). The phosphorylation activates CREB-1, resulting in increased transcription of a number of target genes and their subsequent translation into proteins responsible for the formation of new synaptic connections (Figure 2) (Deisseroth et al., 1996; Ahmed and Frey, 2005; Benito and Barco, 2010).

The signaling pathways outlined above provide a broad understanding about the order and timing of molecular events governing the early and late phase of NMDA-dependent LTP. However, this model lacks information about regulatory changes that might be occurring translationally and post-translationally. In addition, this picture only focuses on a limited number of molecular players and little or no information on their stoichiometry is known. In the next sections, we describe the use of mass spectrometry-based proteomics as a tool to help to elucidate those questions.

## Mass spectrometry (MS)-based proteomics

Proteomics is a system-wide analysis of the proteins expressed in a specific cell, tissue, or organism at a given time (Anderson and Anderson, 1996; Zhang et al., 2013). Although the term proteomics relates to the use of any technology that seeks to interrogate a large number of proteins, it is nowadays used to refer to works where the central platform is mass spectrometry. Currently, the gold standard strategy in MS-based proteomics is shotgun proteomics (Mann and Kelleher, 2008; Domon and Aebersold, 2010). Here, a complex mixture of proteins is digested into peptides with a protease of interest, usually trypsin, which cleaves on the C-terminal side of lysine and arginine. Subsequently, the peptides are separated online by reverse-phase liquid chromatography (LC) and analyzed by mass spectrometers such as quadrupole/time-of-flight (QTOF), ion trap (IT), orbitrap (OT), or ion cyclotron resonance (ICR) (Marshall et al., 1998; Michalski et al., 2011; Thakur et al., 2011; Beck et al., 2015).

**Figure 3:**
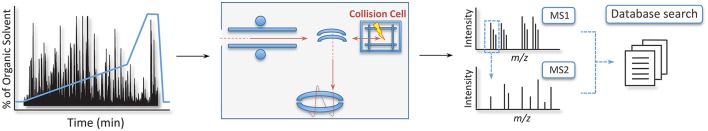
**Workflow of the gold standard strategy in shotgun proteomics**. Here, peptides are separated online in a reverse-phase liquid chromatography and electrosprayed into the mass analyzer. The mass spectrometer measures the peptides' m/z and intensity in the MS1 cycle. Upon fragmentation, the product ions of each peptide are reanalyzed to obtain sequence information of the analyte in the MS2 cycle. Once this experimental data is acquired, information is searched against a database of the organism of interest to identify the proteins in the sample.

In a typical shotgun experiment, the LC-MS/MS run takes up to 120 min and is composed of thousands of cycles, each one made of a MS1 scan—also known as a full scan—that measures the peptides' mass to charge ratio (m/z) and intensity, and a MS2 or MS/MS scan. During the MS2 scan, the 20 most intense peptides in each cycle are fragmented in a collision cell, usually filled with an inert gas such as nitrogen or helium, and their spectra are again measured to obtain sequence information (Geiger et al., 2011; Thakur et al., 2011). Once acquired, the LC-MS/MS data are used in searches against databases containing peptides digested *in silico* to identify the proteins present in the sample(s) (Figure 3) (Sadygov et al., 2004).

**Figure 4:**
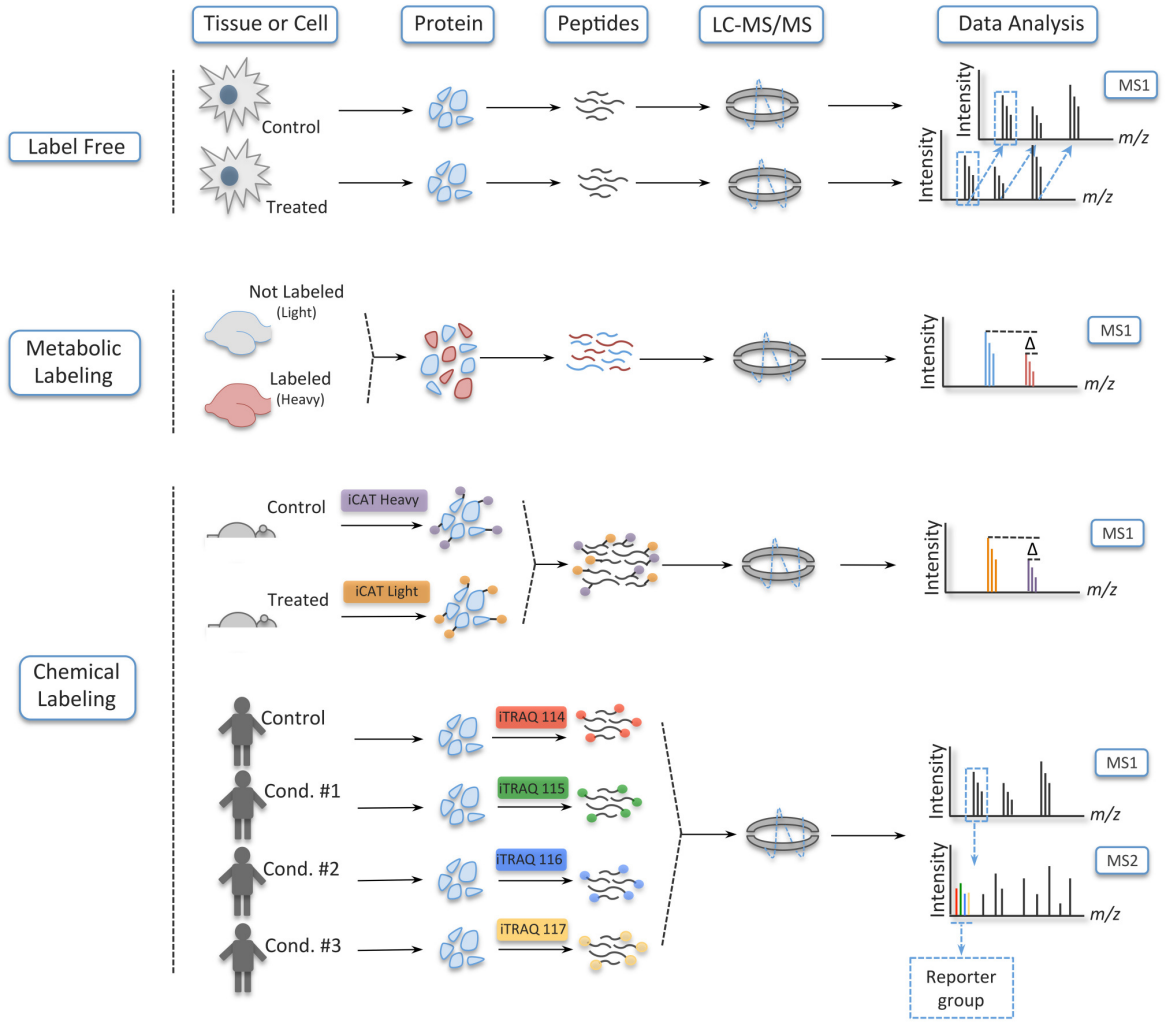
**Different strategies to quantify peptides**. In label free experiments, samples are digested and ran separately in a mass spectrometer; they are combined only in the data analysis. In metabolic labeling, on its turn, one of the conditions is grown in medium containing amino acids labeled with heavy isotopes or heavy nitrogen. Here, the samples are combined very early, and sample handling and analysis are done concomitantly. Lastly, in chemical labeling, tags are incorporated at the protein level, as is the case of iCAT, or at the peptide level with iTRAQ and TMT. In these strategies, the samples are combined early in the workflow of the experiment without increasing the complexity of the samples in the MS1, since the tags of iTRAQ and TMT are isobaric—being distinguished only upon fragmentation in a collision cell.

In addition to protein identification, mass spectrometry can be used to extract quantitative information from samples. Protein quantification can be absolute, if known amounts of a heavy analog of the analyte of interest is added prior to the analysis in a mass spectrometer, or relative, if samples in different states are compared (e.g., brain tissue from rodents trained in a behavioral paradigm vs. controls) (Kettenbach et al., 2011; Bantscheff et al., 2012). The most popular strategies for relative quantification are label-free, metabolic labeling, and chemical labeling (Figure 4).

In label-free quantification, as the name suggests, no label is added to the samples, which are digested and run individually in the mass spectrometer—being the results computationally combined after the acquisition of the data (Filiou et al., 2012). In this strategy, quantification takes advantage of the area plotted over time for each ion as it elutes from the chromatographic column. Later, this extracted ion chromatogram (XIC) is aligned across different samples, and a ratio for each peptide is obtained. Another mode of quantification in label-free experiments is spectrum counting. Here, quantification is based on the number a particular peptide is fragmented during a LC-MS/MS run, which serves as a proxy for abundance and can be compared between conditions (Bantscheff et al., 2007; Hernández et al., 2012). Label-free is regarded as the least accurate strategy of relative quantification, but it has gained momentum due to its low cost, improvements in sample handling, refinement of the chromatographic setup, and development of software for accurate data analysis (Ong and Mann, 2005; Altelaar and Heck, 2012).

By contrast, in metabolic labeling, prior to protein extraction at least one of the conditions is labeled with a heavy stable isotope such as ^15^N or heavy amino acids such as lysine, arginine, or both (Ong, 2002; Rauniyar et al., 2013). The use of a heavy analog prevents the variation usually encountered in label-free experiments, since the samples are mixed, digested and analyzed simultaneously in the LC-MS/MS run. This can be accomplished because heavy (labeled) and light (non-labeled) peptides retain the same physicochemical properties (e.g., retention time during the LC), but a mass shift between them enables their distinction latter in the data analysis. The only exception to this rule is deuterium (^2^H), which is more hydrophilic than hydrogen; this creates a delay in the retention time between the labeled and non-labeled conditions (Yi et al., 2005). Here, as it is also the case in label-free experiments, quantification is acquired by the peak area ratios of the heavy and light peptides in the XIC (Ong, 2002; Bantscheff et al., 2007).

Even though metabolic labeling is the most accurate relative quantification strategy, it has a restricted capacity to multiplexing due to the limitation on the isotopes that can be added to an amino acid and the increase in sample complexity in the MS1 (Hebert et al., 2013). Chemical labeling, in its turn, is able to circumvent those limitations. To this day, many chemical labeling reagents have been developed, but the most used are isotope-coded affinity tags (iCAT), tandem mass tags (TMT), and isobaric tags for relative and absolute quantification (iTRAQ) (Gygi et al., 1999; Thompson et al., 2003; Ross, 2004). iCAT, which labels samples at the protein level, uses the tags that are composed of a reactive group that binds to reduced cysteine residues, a linker group that incorporates isotopes in the heavy reagent, and a biotin affinity group for the isolation of the iCAT-labeled peptides. In a typical experiment using iCAT, the reagent labels protein samples at two different conditions (the light and heavy version of the tags), which are then mixed together and enzymatically cleaved. Next, the peptides with the tags are enriched by avidin affinity chromatography and analyzed in a LC-MS/MS run. Here, quantification is obtained by the peak area ratios of the heavy and light peptides (Yi et al., 2005).

iCAT possesses the same limitation in multiplexing as metabolic labeling, yet iTRAQ and TMT enables from 8 to 10 samples, respectively, to be analyzed in a single experiment. iTRAQ and TMT are isobaric tags that label analytes at the peptide level. Their tags are composed of a reactive group that binds to the N-terminal of peptides and lysine residues, a balance group—which ensures that the same peptides in the different conditions elute together and are indistinguishable in the MS1 scan—and a reporter group. Unlike other quantitative strategies, quantification on iTRAQ and TMT is based on the intensity signal of the reporter group that is released from the analytes upon fragmentation in a collision cell. Nevertheless, chemical labeling also has limitations and some considerations have to be taken to get around these drawbacks (For in depth information on iTRAQ and TMT strategies see Bantscheff et al., 2008; Karp et al., 2010; Ting et al., 2011; Wenger et al., 2011).

## Proteomic studies of memory

The shotgun quantitative strategies described above have fostered a revolution in many fields of biology such as cancer, immunology, and neuroscience by improving our understanding of the systemic cellular response of stimulated or disease states vs. control (Krüger et al., 2008; Dahlhaus et al., 2011; Geiger et al., 2012; Boersema et al., 2013; Meissner and Mann, 2014; Nascimento and Martins-de-Souza, 2015). Yet, when it comes to the proteomic study of memory, very few MS-based experiments have been carried out so far. Some authors believe that such discrepancy is due to the difficulty to characterize proteins that are genuinely associated with this cognitive process due to high biological variability among individuals within areas related to memory such as the hippocampus (Dieterich and Kreutz, 2015). Nevertheless, this explanation falls short, since transcriptomics assays to investigate memory have been conducted before with success (Ponomarev et al., 2010; Bero et al., 2014).

In addition, two recent proteomic studies have demonstrated that those kinds of experiments are feasible. In the first one, Borovok et al. used a radial arm maze (RAM) paradigm to understand the process of memory consolidation in the hippocampus of mice (Borovok et al., 2016). In summary, the RAM behavioral task works as follows: a central circular chamber is connected to eight long arms, which are open to the animal upon the removal of a guillotine door. In the end of four of the arms, for example, visual clues are set to mark where the food are placed. During the experiment, the animals are positioned in the central chamber and learn to associate the visual clues to where the baits are (Götz and Ittner, 2008). Hence, using this paradigm, Borovok and colleagues extracted proteins at different time points during the learning period and quantified those samples by the label-free strategy. They showed the protein abundance profiles during spatial memory formation in the hippocampus, and demonstrated a clear correlation between temporal memory formation and proteins belonging to specific functional groups using gene ontology and signaling pathways analysis.

In the other study, Rao-Ruiz et al. used contextual fear conditioning to understand membrane protein dynamics of mice at 1 and 4 h after training (Rao-Ruiz et al., 2015). Contextual fear conditioning is a form of associative learning, which works as this: mice are placed inside a chamber with a stainless steel grid floor, so that a foot shock can be administered after the mice have explored the box (called delayed-shock paradigm) or as soon as the animal has been placed into the cage (called immediate-shock paradigm) (Rao-Ruiz et al., 2011). Using both training protocols, in addition to untrained control mice, Rao-Ruiz et al., 2015 compared the groups using iTRAQ at 1 and 4 h after conditioning. They found no difference in protein abundance between the experimental groups at 1 h after training; however, 164 proteins in the delayed-shock group and 273 proteins in the immediate-shock group exhibited different abundances at the 4 h time point. Functional protein group analysis of those analytes were carried out, which showed that the most prominent proteins are linked to endocytosis, glutamate signaling and neurotransmitter metabolism (For other proteomic studies on memory see Henninger et al., 2007; Piccoli et al., 2007; Freeman et al., 2009; Zheng et al., 2009; Rosenegger et al., 2010; Li et al., 2011; Monopoli et al., 2011; Kähne et al., 2012; Monje et al., 2012; Lee et al., 2013; Matsuura et al., 2013; Trinidad et al., 2013; Végh et al., 2014; Zhang et al., 2014).

## Targeted proteomics

The shotgun strategy has been considered the gold standard approach to identify and quantify proteins in complex proteomes (Aebersold and Mann, 2003; Smith et al., 2013). However, this discovery-based method has inherently poor reproducibility due to its stochastic selection of parent ions and its modest dynamic range, which renders the information acquired incomplete or biased toward a subset of proteins (Domon and Aebersold, 2006). These drawbacks have lead to the maintenance of immunochemical assays such as antibody arrays and ELISA—techniques that quantitatively measure a small set of targets with high accuracy and reproducibility—very popular in the scientific community (Edwards et al., 2011). However, advancements in targeted proteomic approaches such as selected reaction monitoring (SRM), also known as multiple reaction monitoring (MRM), will likely change this scenario (Picotti et al., 2013).

Selected reaction monitoring (SRM) is a strategy that dates back to the development of quadrupole mass analyzers (Yost and Enke, 1979). However, only in the last few years this method has reached its full potential as a high precision analytical tool (Kuhn et al., 2004). As in shotgun proteomics, SRM uses peptides as surrogates to protein identification and quantification, but this approach is not a discovery assay. SRM is a hypothesis-driven strategy like other classical techniques, and prior information about the analytes being interrogated need to be known, regardless if its sources are from previous discovery experiments, proteomic data repositories such as PeptideAtlas and PRIDE or predicted by using computational algorithms (Deutsch et al., 2008; Jones et al., 2008; Lange et al., 2008; Gallien et al., 2011; Picotti and Aebersold, 2012).

**Figure 5:**
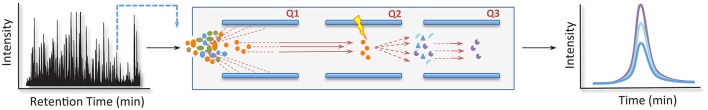
**Selected reaction monitoring (SRM) pipeline**. In a typical SRM experiment, a triple quadrupole instrument is used. Here, the first and third mass analyzers, also known as Q1 and Q3, respectively, function as ion filters, selecting only the analytes with a specific m/z value at a predetermined retention time. The second quadrupole (Q2), in its turn, works as a collision cell to fragment the parent ions selected in the Q1. In this strategy, quantification *per-se* is obtained through the peak area ratios of the transition ions from an endogenous peptide and a reference analog.

The classical SRM experiment is carried out in triple quadrupole instruments, where the three analyzers are aligned in tandem. The first and third mass analyzers, also known as Q1 and Q3, respectively, function as ion filters, selecting only the analytes with a specific m/z value. The second quadrupole (Q2), on the other hand, is used as a collision cell to fragment the parent ions selected in the Q1; the combination of parent ion and its fragments is called a “transition” (Figure 5) (Gallien et al., 2011). In addition, such experiments usually use an isotopic-labeled internal analog for all the peptides under investigation. This confers SRM assays quality control for the sample preparation procedures as well as compensation for discrepancies that can arise through ion suppression and spray instability (Brun et al., 2007; Carr et al., 2014). Quantification *per-se* is obtained through the peak area ratios of the transition ions from the endogenous peptide and its heavy analog (Brun et al., 2007).

Those features confer targeted proteomics with the high accuracy and reproducibility usually required in the biological and clinical sciences. A number of recent studies have taken advantage of the power of SRM to provide a deeper understanding of mechanisms and structures related to memory (Munton et al., 2007; Trinidad et al., 2013; Lowenthal et al., 2015). For example, Cheng et al. compared purified postsynaptic densities of the forebrain and cerebellum (Cheng et al., 2006). Employing the ICAT strategy, they identified 43 proteins displaying statistically significant abundance change between the PSDs and monitored 32 of them using synthetic isotopic-labeled analogs in a SRM pipeline. Measuring their molar abundances, Cheng et al. demonstrated that these postsynaptic regions possess unexpected stoichiometric differences, something that might also be true to hippocampal PSDs.

## Conclusion

The study of the molecular mechanisms of memory is an area of great interest to the scientific community, where the first studies date back to Santiago Ramón y Cajal and the experimental psychologists such as the behaviorists (Moser et al., 2015). Nonetheless, much of the work carried out until the present time has concentrated in the use of classical methods of molecular biology, genetics, and biochemistry, which target a limited number of analytes, and are time consuming and laborious. On that account, we have a restricted understanding about the processes that happen in the brain cells and how they are related to one another during memory formation (Dieterich and Kreutz, 2015).

Shotgun and targeted proteomics approaches might be able to speed up this process. Discovery proteomics allows thousands of proteins, including PTMs such as phosphorylation, to be identified and quantified in a single experiment (Wu et al., 2011; Weekes et al., 2014; Humphrey et al., 2015; Peshkin et al., 2015; Sharma et al., 2015; Wühr et al., 2015). The SRM strategy, on its turn, is able to determine with high accuracy and reproducibility the stoichiometry of proteins in a sample, multiplexing up to 100 peptides per experiment (Kuhn et al., 2011; Ebhardt et al., 2015). Moreover, as the number of discovery experiments increase, improving our understanding of the molecular players involved in memory formation, more accurate measurements will be required to develop models to understand how proteins work together. Hence, we expect that soon targeted proteomics will become the flagship of MS-based proteomics.

## Author contributions

All authors listed, have made substantial, direct and intellectual contribution to the work, and approved it for publication.

### Conflict of interest statement

The authors declare that the research was conducted in the absence of any commercial or financial relationships that could be construed as a potential conflict of interest.
